# Treatment-related skin reactions in enfortumab vedotin as a surrogate marker of survival and treatment response

**DOI:** 10.1007/s10147-024-02672-3

**Published:** 2024-12-16

**Authors:** Jun Nagayama, Satoshi Inoue, Hiroki Sai, Akira Hayakawa, Yuri Yuguchi, Tomohide Suzuki, Hirotaka Matsui, Takuma Yuba, Koya Morishita, Shusuke Akamatsu

**Affiliations:** 1 Department of Urology, Nagoya University Graduate School of Medicine, 65 Tsuruma-cho, Showa-Ku, Nagoya, Aichi 466-8550 Japan; 2 Department of Urology, Japanese Red Cross Aichi Medical Center Nagoya Daiichi Hospital, Nagoya, Japan; 3 Department of Urology, Yokkaichi Municipal Hospital, Yokkaichi, Japan; 4 Department of Urology, Chukyo Hospital, Nagoya, Japan; 5 Department of Urology, Nagoya Medical Center, Nagoya, Japan; 6 Department of Urology, Toyohashi Municipal Hospital, Toyohashi, Japan; 7 Department of Urology, Kariya Toyota General Hospital, Kariya, Japan; 8 Department of Urology, Komaki Municipal Hospital, Komaki, Japan

**Keywords:** Enfortumab vedotin, Skin rash, Adverse effects, Bladder cancer, Urothelial carcinoma

## Abstract

**Background:**

Treatment-related skin reactions (TRSRs) induced by enfortumab vedotin (EV) targeting nectin-4 are among the most common adverse events. However, their association with survival and treatment response is poorly understood.

**Methods:**

We retrospectively identified patients who received EV from December 2021 to April 2023 at Nagoya University Hospital and its affiliated facilities and extracted clinical data from their medical records. We evaluated cancer-specific survival (CSS) and progression-free survival (PFS) as survival outcomes and overall response rate (ORR) and disease control rate (DCR) as treatment responses between patients with and without TRSRs.

**Results:**

In total, 67 eligible patients were identified. Thirty-four patients experienced TRSRs, and the remaining 33 did not experience TRSRs. The median follow-up period was 8 months. Patients in the TRSRs group demonstrated significantly longer median CSS (15 vs. 8 months; *p* = 0.003) and median PFS (10 vs. 5 months;* p* < 0.001) than the non-TRSRs. Regarding treatment response, the patients in the TRSRs group showed a favorable, albeit nonsignificant, treatment response trend compared with those in the non-TRSRs group (ORR, 73.5% vs. 51.5%; *p* = 0.107; DCR, 91.2 % vs. 81.8%; *p =* 0.444).

**Conclusions:**

Patients with TRSRs demonstrated more prolonged survival and superior treatment responses to EV treatment. The role of TRSR as a surrogate marker of EV’s efficacy should be further explored in prospective and sufficiently powered studies.

**Supplementary Information:**

The online version contains supplementary material available at 10.1007/s10147-024-02672-3.

## Introduction

In Japan, enfortumab vedotin (EV) was approved in November 2021 as a third-line therapy for patients with advanced urothelial carcinoma (UC) that is refractory to prior platinum-based chemotherapy and immune-checkpoint inhibitors (ICIs) such as programmed cell death 1 (PD-1) or PD-1 ligand 1 (PD-L1) inhibitors [1, 2]. EV is a novel antibody-drug conjugate (ADC) targeting nectin-4 expressed in patients with UC comprising an anti-nectin-4 antibody, protease-cleavable linker, and microtubule-disrupting agent monomethyl auristatin E (MMAE) [3, 4]. These elements work sequentially and specifically in cells expressing nectin-4, consequently inducing apoptosis and cell death [3, 5].

Nectin-4 is a Ca^2+^-independent immunoglobulin-like cell-cell adhesion molecule generally expressed in human epidermal keratinocytes and skin appendages [3, 5]. The positive expression rate in normal skin exceeded 90% [6]. Therefore, treatment-related skin reactions (TRSRs) are frequently occurring adverse events (AEs) [1, 4]. Meanwhile, nectin-4 is also expressed in several carcinomas and is associated with tumor progression [3, 7]. Especially in UC, high-frequency nectin-4 expression is observed across the disease spectrum (87% in the bladder, 66% in the upper urinary tract, and 92% in metastatic sites) [3, 8].

Despite high expression of nectin-4 in the skin, TRSRs are observed in only about 55% of patients, suggesting that susceptibility to TRSRs varies even among those expressing nectin-4 [1, 9, 10]. We hypothesized that the disparities in susceptibility to TRSRs may also impact UC’s response to EV. According to this theory, TRSRs could serve as a significant predictive indicator of EV; [11] however, the association between TRSRs and treatment response or survival benefits has not been fully discussed.

Therefore, we evaluated how TRSRs to EV are associated with treatment response and survival outcomes in patients with advanced UC receiving EV as the third- or later-line treatment.

## Patients and methods

### Patient population

This retrospective multicenter collaborative study was conducted as part of the overarching MEGUMI (MEidai GenitoUrinary Mega Investigation) project. Nagoya University Hospital and its affiliated hospitals conduct clinical research and share a research database. The entire study was centrally approved by the institutional review board of Nagoya University Graduate School of Medicine (approval number: 2016-0474-5). Individual IRB approval at the affiliate hospitals was officially waived.

**Fig. 1:**
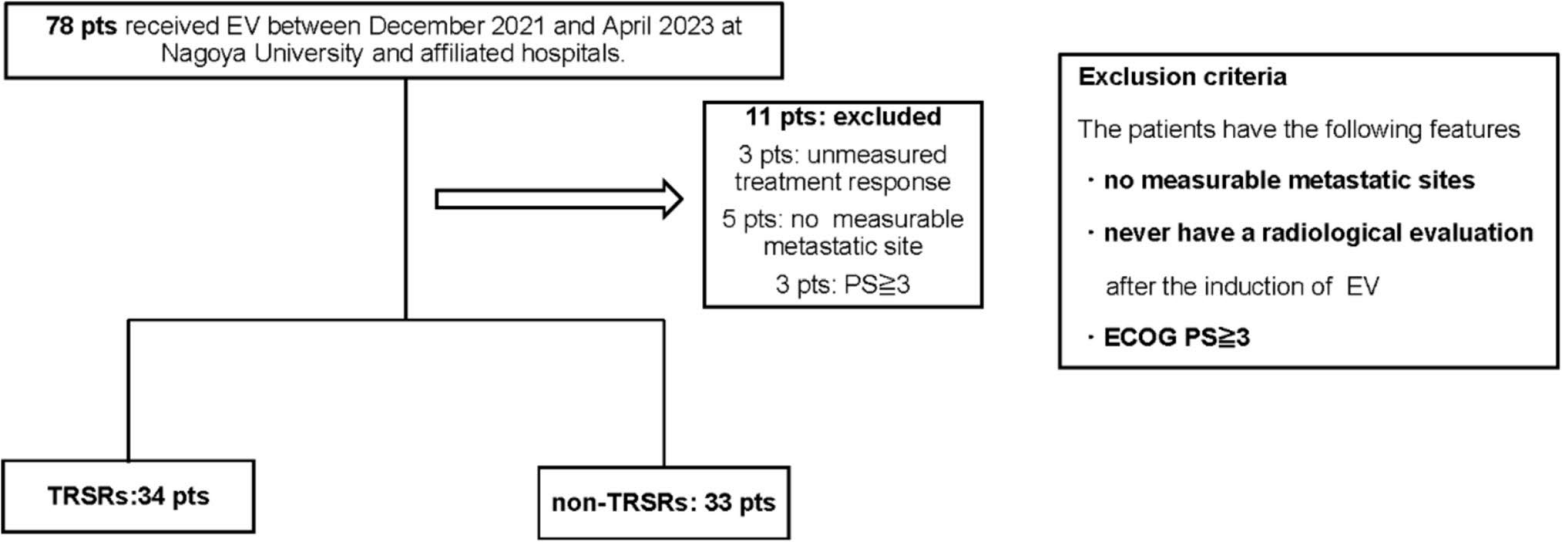
Enrolled patients’ inclusion criteria

We identified 78 patients who received EV between December 2021 and April 2023 at Nagoya University Hospital and seven affiliated hospitals. All the enrolled patients underwent radiographical evaluation at least once after EV initiation. We excluded those with no evaluable metastatic sites and Eastern Cooperative Oncology Group performance statuses (ECOG PS) ≥3. The remaining 67 patients were classified into two groups based on the TRSR occurrence. The flowchart explaining patient inclusion is shown in Figure 1.

### Treatment and evaluation

EV was administered at 1.25mg/kg on days 1, 8, and 15 of a 28-day cycle. Each physician’s decision based on the patient’s status allowed an initial dose reduction at EV initiation. The physicians also determined if treatment would be interrupted or discontinued based on the recommended dosing criteria of EV, patients’ general status, physical findings, radiological findings, and laboratory data at each visit. All enrolled patients received at least one radiological evaluation by computed tomography, combined with magnetic resonance imaging in some cases. The radiological assessment interval depended on physicians’ policy, which ranged from one to three months. The treatment response was assessed based on Response Evaluation Criteria in Solid Tumors (RECIST) version 1.1 [12].

### Data extraction

We carefully examined the medical records of the enrolled patients and gathered detailed patient demographic information, including age; sex; ECOG PS; comorbidities; primary and metastatic tumor locations; details of prior treatments; treatment details of EV, such as treatment cycles, dose reduction, and treatment discontinuation due to treatment-related adverse events (TRAEs); laboratory data; and pathology data. In addition, we computed the Bellmunt risk score using the patients’ demographic details [13]. TRSRs were assessed for onset time, grade, dose reduction and treatment discontinuation due to TRSRs. We also evaluated the treatment for TRSRs, and skin response manifestations. All TRAEs including TRSRs were graded according to the Common Terminology for Adverse Events (CTCAE) version 5.0 [14].

### Outcome measures

First, we investigated the relationship between the TRSR occurrences and survival outcomes, in which cancer-specific survival (CSS) and progression-free survival (PFS) were evaluated. CSS was defined as the duration from the EV initiation to cancer-specific death. PFS was defined as the duration from the EV initiation to confirmation of disease progression or death.

Then, we assessed the relationship between the TRSRs and treatment response. The best response was defined according to RECIST. We calculated the overall response rate (ORR) and disease control rate (DCR) based on the data. ORR was the sum of the partial response (PR) rate and complete response (CR) rate, and DCR also included those with stable disease (SD).

### Statistical analysis

Chi-square and Mann–Whitney U tests were used to compare categorical and continuous variables and evaluate the patients’ demographic data. Categorical variables are reported as frequencies and percentages. Furthermore, continuous variables are reported as averages with standard deviations (SD) for normal distribution and medians with interquartile ranges (IQR) for non-normal distribution, respectively.

We estimated CSS and PFS used the Kaplan–Meier method and between-group comparisons, using the log-rank test to calculate the groups’ median survival and 95 % confidence interval (CI). Furthermore, the Chi-squared test compared ORR and DCR between the two groups.

Finally, multivariable Cox proportional hazard model analysis was performed to assess the prognostic factors for CSS and PFS, including age, sex, ECOG PS, Bellmunt risk score, diabetes, primary site, metastatic site, liver metastasis, line of therapy, most recent systemic therapy before EV, dose reduction/treatment discontinuation, prior radical therapy, and TRSRs.

All statistical analyses were performed using the EZR software (Saitama Medical Center, Jichi Medical University, Saitama, Japan), a graphical user interface for R software (R Foundation for Statistical Computing, Vienna, Austria) [15]. All *p-*values are two-sided, and *p* <0.05 was considered statistically significant.

## Results

### TRSRs

In the present study, TRSRs occurred in 50.7% of EV. Regarding TRSR type, erythema—including erythema multiform—occurred in 32.4%, followed by bullous dermatitis in 11.8%. Eighty percent of TRSRs occurred within three cycles of EV treatment, and most were low-grade (Grade 1–2). TRSRs affected dose intensity, and 50% (17/34) of the patients with TRSRs experienced at least either dose reduction or treatment discontinuation. Moreover, 17.6% (3/17) and 41.2% (7/17) of these patients experienced two-step dose reduction and treatment discontinuation, respectively. The details on TRSRs are shown in Table 1.

**Table 1:** Detailed treatment-related skin reaction data

Variables	TRSRs (n = 34)
Onset time of TRSRs, no. (%)	
1–3 cycles	27 (79.4)
≥4 cycles	7 (20.6)
CTCAE grade, no. (%)	
1–2	27 (79.4)
3	7 (20.6)
Type of TRSRs, no. (%)	
Erythema	9 (26.5)
Erythema multiforme	2 (5.9)
Bullous dermatitis	4 (11.8)
Rash with details unknown	19 (55.9)
Dose reduction of EV due to TRSRs, no. (%)	12 (35.3)
One-step dose reduction (1.0 mg/kg), no. (%)	9
Two-step dose reduction (0.75 mg/kg), no. (%)	3
Discontinuation EV due to TRSRs, no. (%)	7 (20.6)
Treatment, no. (%)	
Topical corticosteroid	28 (82.4)
Systemic corticosteroid	3 (8.8)
Antihistamine	11(32.4)

*CTCAE* Common Terminology Criteria for Adverse Events, *EV* enfortumab vedotin, *TRSRs* treatment-related skin reactions

### Patients’ characteristics

Sixty-seven patients were identified; their characteristics are shown in Table 2. At baseline, there were no remarkable differences in the distribution of patients’ ages, sex, and ECOG PSs between the TRSRs and non-TRSRs groups. The variance among the two groups was present in the Bellumunt risk score (≥2: 32.4% vs. 51.5% in the TRSRs and non-TRSRs groups) and primary site (bladder: 52.9% vs. 63.6%; upper urinary tract: 41.2% vs. 21.2%; bladder and upper urinary tract: 5.9% vs. 15.2%); however, these differences did not rise to the level of statistical significance. Regarding laboratory data, only hemoglobin level was higher in the TRSR group than in the non-TRSRs group, whereas no differences were noted in the other variables (Supplementary Table 1). Patients in the non-TRSRs group were more likely to have received chemotherapy (8.8% vs. 27.3%; *p* = 0.099) and received more chemotherapy cycles; however, the median cycles were similar between the groups (median 4 cycles; IQR 2–4 cycles vs. median 4 cycles; IQR 3.75–6 cycles; *p* = 0.041). Prior ICI reception was also similar between the two groups. Moreover, the TRSRs group received more EV cycles than the non-TRSRs group (median 6 cycles; IQR 4–11 cycles vs. median 4 cycles; IQR 2–6 cycles; *p* = 0.004).

**Table 2:** Patients’ characteristics

Variables	TRSRs (n = 34)	Non-TRSRs (n = 33)	*p*
Age median, years (IQR)	71.5 (64.75–75.75)	73.0 (68.0–75.0)	0.546
Sex, no. (%)			
Male	26 (76.5)	27 (81.8)	0.812
Female	8 (23.5)	6 (18.2)	
ECOG PS, no. (%)			
0–1	32 (94.1)	29 (87.9)	
2	2 (5.9)	4 (12.1)	0.641
Bellmunt risk score^a^, no. (%)			
0–1	23 (67.6)	16 (48.5)	0.180
≥2	11 (32.4)	17 (51.5)	
Diabetes, no. (%)	10 (29.4)	5 (15.2)	0.268
Primary site, no. (%)			
Bladder	18 (52.9)	21 (63.6)	0.147
Upper urinary tract	14 (41.2)	7 (21.2)	
Bladder + upper urinary tract	2 (5.9)	5 (15.2)	
Metastatic site, no. (%)			
Lymph node only	9 (26.5)	10 (30.3)	0.939
Visceral	25 (73.5)	23 (69.7)	
Liver	10 (29.4)	8 (24.2)	0.840
Line of therapy, no. (%)			
3	26 (76.5)	22 (66.7)	0.536
≥4	8 (23.5)	11 (33.3)	
The most recent systemic therapy before EV, no. (%)			
ICI	31 (91.2)	24 (72.7)	0.099
Chemotherapy	3 (8.8)	9 (27.3)	
Prior chemotherapy, cycles (IQR)	4 (2–4)	4 (3.75–6)	**0.041**
Prior ICI, cycles (IQR)	6 (4–12)	5 (4–12)	0.670
Duration of prior therapy to EV, months (IQR)	0 (0–1)	0 (0–1)	0.611
EV, cycles (IQR)	6 (4–11)	4 (2–6)	**0.004**
Dose reduction due to TRAEs, no. (%)	12 (35.3)	5 (15.2)	
One-step reduction (1.0mg/kg)	9	4	0.107
Two-step reduction (0.75mg/kg)	3	1	
Discontinuation due to TRAEs, no. (%)	7 (20.6)	3 (9.1)	0.328
Prior radical therapy, no. (%)			
Yes	21 (61.8)	19 (57.6)	0.920
No	13 (38.2)	14 (42.4)	
Variant histology, no. (%)	1 (4.2)	2 (7.7)	1.000

*IQR* interquartile range, *ECOG PS* Eastern Cooperative Oncology Group performance status, *EV* enfortumab vedotin, *ICI* immune-checkpoint inhibitor, *TRAEs* treatment-related adverse events

^a^Bellmunt risk score-liver metastases, hemoglobin level < 10g/dL, and ECOG PS > 0 are the factors; every point is added for each factor

### Survival and treatment response in comparison with and without TRSRs

**Fig. 2:**
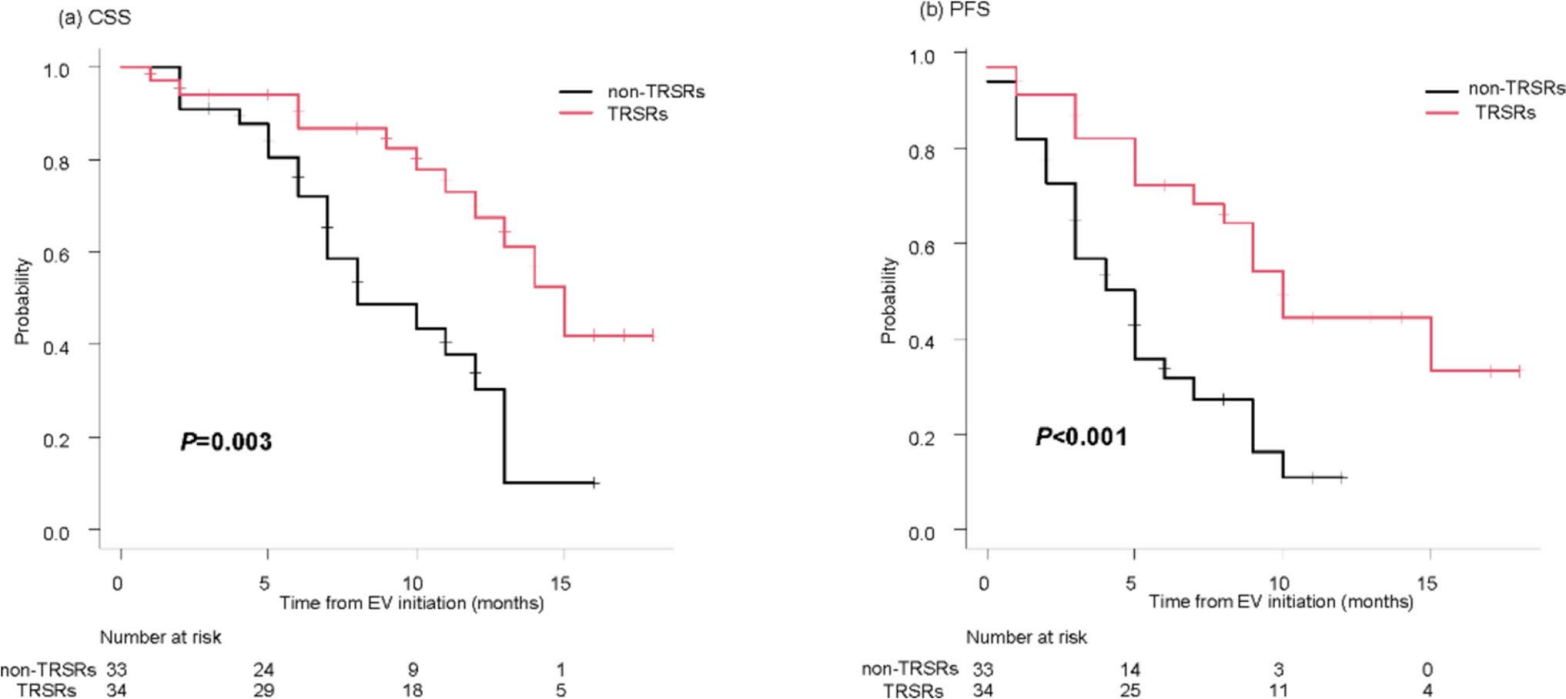
Kaplan–Meier curves showing the comparison of CSS and PFS between patients with and without TRSRs

In the overall cohort, the median follow-up was 8 months (IQR: 5–12), and the median CSS and PFS were 13 (95% CI: 10–15) and 7 months (95% CI: 5–9), respectively. The TRSRs group demonstrated significantly longer median CSS compared to the non-TRSRs group (15 months [95% CI: 12–not available (NA)] vs. 8 months [95%CI: 7–13]; *p =* 0.003). Moreover, the TRSRs group also showed significantly longer median PFS (10 months [95% CI: 7–NA] vs. 5 months [95%CI: 3–6];* p*<0.001). The Kaplan–Meier curves for CSS and PFS are shown in Figure 2. No patients demonstrated a CR. The TRSRs group showed higher ORR and DCR. However, there was no statistical significance between-group differences (ORR: 73.5% vs. 51.5%; *p* = 0.107, DCR: 81.8% vs. 91.2%; *p =* 0.444; Table 3). Multivariable Cox proportional hazard analysis was also performed for survival outcomes. TRSRs were shown as a favorable prognostic factor in CSS and PFS (CSS, HR 0.45 [95% CI, 0.20, 1.05]; *p* = 0.064; PFS, HR 0.48 [95% CI, 0.24, 0.96];* p* = 0.039), although no significant difference was found by a slight margin in CSS. These results are shown in Table 4.

**Table 3:** Comparison of treatment response between the TRSR group and non-TRSR group

Variables	Overall (n = 67)		
Best response, no (%)			
CR	0 (0.0)		
PR	42 (62.7)		
SD	16 (23.9)		
PD	9 (13.4)		
ORR, %	62.7		
DCR, %	86.6		
Variables	TRSRs (n = 34)	Non-TRSRs (n = 33)	*p*
Best response, no (%)			
CR	0 (0.0)	0 (0.0)	
PR	25(73.5)	17(51.5)	
SD	6 (17.6)	10(30.3)	
PD	3 (8.8)	6 (18.2)	
ORR, %	73.5	51.5	0.107
DCR, %	91.2	81.8	0.444

*CR* complete response, *PR* partial response, *SD* stable disease, *PD* progressive disease, *ORR* overall response rate, *DCR* disease control rate, *TRSRs* treatment-related skin reactions

**Table 4:** Univariate and multivariable Cox proportional hazard model analysis for CSS and PFS

	Univariate (HR, 95% CI)	p value	Multivariable (HR, 95% CI)	p value
*CSS*				
Age				
≥72 vs. <72	2.06 (0.94–4.50)	0.071		
Sex				
Male vs. female	0.87 (0.35–2.15)	0.764		
ECOG PS				
0–1 vs. 2	2.60 (0.98–6.90)	0.054		
Bellmunt risk score^a^				
0–1 vs. ≥2	1.64 (0.79–3.41)	0.183		
Diabetes				
No vs. yes	1.60 (0.68–3.76)	0.287		
Primary site				
Bladder (ref)				
Upper urinary tract	0.92 (0.39–2.18)	0.851		
Bladder + upper urinary tract	1.74 (0.63–4.76)	0.285		
Metastatic site				
LN vs. visceral	0.54 (0.25–1.18)	0.121		
Liver metastasis				
No vs. yes	0.60 (0.26–1.39)	0.237		
Line of therapy				
3 vs. ≥4	1.39 (0.66–2.95)	0.386		
The most recent systemic therapy before EV				
Chemotherapy vs. ICI	0.61 (0.27–1.38)	0.233		
Dose reduction/discontinuation				
No vs. yes	0.33 (0.13–0.82)	**0.017**	0.47 (0.18–1.27)	0.139
Prior radical therapy				
No vs. yes	0.77 (0.37–1.60)	0.484		
TRSRs				
No vs. yes	0.33 (0.15–0.72)	**0.005**	0.45 (0.20–1.05)	0.064
*PFS*				
Age				
≥72 vs. <72	1.25 (0.67–2.35)	0.489		
Sex				
Male vs. female	1.10 (0.46–2.64)	0.823		
ECOG PS				
0–1 vs. 2	2.43 (1.02–5.80)	**0.046**	1.66 (0.67–4.07)	0.272
Bellmunt risk score^a^				
0–1 vs. ≥2	1.48 (0.79–2.76)	0.219		
Diabetes				
No vs. yes	1.16 (0.55–2.44)	0.694		
Primary site				
Bladder (ref)				
Upper urinary tract	0.80 (0.39–1.66)	0.555		
Bladder + upper urinary tract	2.20 (0.88–5.49)	0.091		
Metastatic site				
LN vs. visceral	1.05 (0.53–2.11)	0.884		
Liver metastasis				
No vs. yes	0.90 (0.45–1.79)	0.763		
Line of therapy				
3 vs. ≥4	1.41 (0.74–2.67)	0.291		
The most recent systemic therapy before EV				
Chemotherapy vs ICI	0.56 (0.27–1.14)	0.108		
Dose reduction/discontinuation				
No vs. yes	0.38 (0.19–0.77)	**0.007**	0.54 (0.25–1.15)	0.108
Prior radical therapy				
No vs. yes	0.86 (0.46–1.60)	0.634		
TRSRs				
No vs. yes	0.37 (0.19–0.70)	**0.003**	0.48 (0.24–0.96)	**0.039**

^†^Bellmunt risk score-liver metastases, hemoglobin level < 10g/dL, and ECOG PS > 0 are the factors; every point is added for each factor.

*CI* confidence interval, *HR* hazard ratio, *CSS* cancer-specific survival, *PFS* progression-free survival, *ECOG PS* Eastern Cooperative Oncology Group performance status, *EV* enfortumab vedotin, *ICI* immune-checkpoint inhibitor, *LN* lymph node, *TRSRs* treatment-related skin reactions

### Comparison of survival between patients with and without dose reduction treatment discontinuation of EV

The effect of dose reduction/discontinuation of EV due to any TRAEs was evaluated in all patients. Patients with or without dose reduction/discontinuation were compared, and patients’ characteristics are shown in Supplementary Table 2. The reception cycles of EV and the percentage of grade ≥3 TRAEs were higher in the dose reduction/discontinuation group (median 6 cycles; IQR 4–11 cycles vs. median 4 cycles; IQR 2–7 cycles; *p* = 0.057, Grade ≥3 TRAEs: 36.0% vs. 14.3%; *p* = 0.067), although the other variables were comparable between the groups. The Kaplan–Meier curves for CSS and PFS are shown in Supplementary Figure 1, and the dose reduction/discontinuation group demonstrated significantly longer median survival (CSS: 15 [14–NA] vs. 11 months [7–13]; *p* = 0.010, PFS: 10 [6–NA] vs. 5 months [3–9] ;* p* = 0.003).

We also evaluated how dose reduction/discontinuation due to TRSRs influenced survival outcomes. Patients with dose reduction/discontinuation due to TRSRs were also compared, and patients’ characteristics are shown in Supplementary Table 3. The EV cycles and the occurrence of grade 3 TRSRs were higher in the TRSRs group (median 10 cycles; IQR 6–11 cycles vs. median 5 cycles; IQR 4–9cycles; *p* = 0.097; Grade 3 TRSRs: 35.3% vs. 5.9%; *p* = 0.085). Regarding the other variables, similar characteristics were noted. The Kaplan–Meier curves for CSS and PFS are shown in Supplementary Figure 2. The dose reduction/discontinuation group indicated longer survival than the non-reduction/discontinuation group (CSS: not reached [14–NA] vs. 12 months [6–NA]; *p* = 0.096, PFS: not reached [9–NA] vs. 7 months [3–NA];* p* = 0.020)

## Discussion

EV is a novel therapy for UC refractory to prior chemotherapy and ICIs, which targets nectin-4 expressed in UC [1–3]. Nectin-4 is also expressed homogenously in normal skin keratinocytes and appendages. TRSRs are one of EV’s most frequent AEs [1, 3–5], yet their association with EV survival and treatment response remains uncertain. We compared survival outcomes and treatment response to EV between those with and without TRSRs. The present study demonstrated that patients with TRSRs showed significantly longer CSS and PFS and superior ORR and suggested that susceptibility to EV is shared in skin and UC within an individual patient. Further, TRSRs may serve as a surrogate marker of treatment efficacy.

A Japanese subgroup analysis of EV 301 and real-world data showed median OS and PFS of 10–15 and 4–7 months, respectively [9, 16, 17]. ORR is reportedly 34.4%–57.7% [9, 16], similar to our study (although our cohort showed slightly higher ORR). Several Japanese studies have reported 29.4–55.6% TRSR occurrences [9, 16, 18]. Similarly, approximately half of our patients experienced TRSRs.

TRSRs typically occur relatively soon after EV initiation [1, 19, 20]. Eighty percent of TRSRs in the present cohort happened within the first three cycles, comparable to the previous studies. A study from a single center reported the association between treatment efficacy and TRSRs [21]. The study also showed that the patients with TRSRs showed a significantly superior response (ORR 57.7% vs. 24.0%) [21]. However, the researchers did not examine survival benefits. This was the pioneering study demonstrating an association between TRSRs and survival outcomes.

The mechanism of TRSRs to EV has not been fully elucidated; however, there are two proposed mechanisms. One is the direct delivery of MMAE, which causes damage to normal tissue, such as the epidermis, due to its expression of nectin-4 [4]. Another mechanism could be an allergy-related skin reaction, with type IV delayed hypersensitivity considered its pathology. This is believed to be linked to severe skin reactions such as Stevens-Johnson syndrome and toxic epidermal necrolysis [22]. MMAE’s direct effects are considered to be the leading cause of TRSRs.

In addition, the pathophysiology of EV-related TRSRs has been assessed in several previous studies. Nectin-4, the target of EV, is typically expressed in the human skin [6, 23]. Accordingly, the skin is likely to be affected by the pharmacological effect of EV. Furthermore, biopsy specimens of skin collected from the TRSR group demonstrated prominent ring and starburst mitoses and dyskeratosis within the epidermis, which reflect mitotic activity arrest [24]. These findings were similar to those of taxane-induced toxic erythema [25]. The MMAE of EV and taxanes is the microtubule inhibitor, which promotes the antitumor effect through mitosis inhibition and consequent apoptosis [3, 25]. Therefore, this pharmacological mechanism of EV may be related to TRSRs as well as the antitumor effect.

Similarly, TRSRs due to other antitumor agents have been previously reported as clinical indicators of treatment efficacy. For example, skin reactions related to epidermal growth factor receptor (EGFR) tyrosine kinase inhibitors (EGFR-TKI) for non-small cell lung cancer indicated treatment efficacy [26]. Similar to nectin-4, EGFR is abundantly expressed in the keratinocytes in the epidermis and drives cell cycle activation and proliferation by activating downstream pathways [27, 28]. Accordingly, EGFR-TKI affects keratinocytes and causes TRSRs by inducing growth arrest and apoptosis, which also promote antitumoral effects [28]. Thus, TRSRs due to EGFR-TKI would be induced by the common pharmacological effect that acts in tumors, so the TRSRs could be a clinical indicator of therapeutic effect. In this perspective, EVs and EGFR-TKIs are similar in that their inherent pharmacological effects appear to arise from a common mechanism for AEs and antitumor effects. Therefore, this precedent would support the association between TRSRs and treatment response in EV.

Regarding the association between other TRAEs of EV and treatment efficacy, several studies have reported that peripheral neuropathy (PN) was a favorable factor in survival outcomes [29, 30]. EV-related PN would be MMAE-mediated inhibition of microtubule-dependent axonal transport [31]. However, PN has a relatively late onset, with a median of 15 weeks [30]. Considering this, PN would be a dose-dependent AE, which could affect only responders to EV. Therefore, PN might be a consequent manifestation of sustained therapeutic effect rather than a predictive factor for treatment efficacy.

One possible mechanism explaining the association between treatment response and TRSRs in an individual is genetic polymorphism. ATP binding cassette (ABC) transporter, which is encoded by the multidrug resistance (MDR) genes, promotes the efflux of MMAE [32]. Of these transporters, many single nucleotide variants (SNVs) have been described, and some SNVs have reportedly affected prognosis and AEs in various cancer types [32].

For instance, the specific genotype of rs1045642 variant in *MDR1* was reportedly a favorable prognostic indicator for colorectal cancer treated with oxaliplatin [33]. Regarding ADCs, *MDR1* upregulation was related to poor prognosis in patients with Hodgkin lymphoma who received brentuximab vedotin, an ADC for CD30 incorporated MMAE; however, it was not referred to as whether specific SNVs of ABC transporter regarding MMAE [34]. Thus far, whether the specific variant of *MDR* for MMAE exists remains uncertain, and further investigation is needed.

Furthermore, we evaluated how dose reduction/discontinuation of EV affects treatment efficacy. In this study, 37% of the patients (25/67) experienced dose reduction/discontinuation due to TRAEs. Among these patients, 68% (17/25) had TRSRs. Nevertheless, the dose reduction/discontinuation group demonstrated superior survival outcomes. A Japanese real-world study did not find a relationship between dose intensity and survival outcomes [35]. Rather, several studies that have shown that even lower dose intensity due to PN revealed favorable survival outcomes and response [29, 30]. These results show that dose intensity may not influence survival outcomes.

There are several limitations to the present study. First, it was retrospective and included a small number of patients. Second, there might be unrecognized confounding factors for survival outcomes, although we performed comprehensive analyses. Third, it was a multicenter study, and a central review regarding radiological evaluation was not performed. Finally, TRAEs were based on physicians’ reports, so there might be slight variances regarding grade evaluation. Considering these limitations, further investigation with a larger sample size would be needed to confirm the propriety of our results and verify our hypothesis.

## Conclusions

In the present multicenter, retrospective study, the patients with TRSRs showed superior treatment response and more prolonged survival among those who received EV. TRSR might be a reasonable surrogate marker of treatment efficacy and predict good prognosis in the patients treated with EV.

## Supplementary Information

Below is the link to the electronic supplementary material.

Supplementary file1 (DOCX 240 KB)

## Data Availability

The data that support the findings of this study are available on request from the corresponding author, Jun Nagayama. The data are not publicly available due to the containing information that could compromise the privacy of research participants.

## References

[1] Powles T, Rosenberg JE, Sonpavde GP et al (2021) Enfortumab vedotin in previously treated advanced urothelial carcinoma. N Engl J Med 384:1125–1135 33577729 10.1056/NEJMoa2035807 PMC8450892

[2] Chang E, Weinstock C, Zhang L et al (2021) FDA approval summary: enfortumab vedotin for locally advanced or metastatic urothelial carcinoma. Clin Cancer Res 27:922–927 32962979 10.1158/1078-0432.CCR-20-2275

[3] Challita-Eid PM, Satpayev D, Yang P et al (2016) Enfortumab vedotin antibody-drug conjugate targeting nectin-4 is a highly potent therapeutic agent in multiple preclinical cancer models. Cancer Res 76:3003–3013 27013195 10.1158/0008-5472.CAN-15-1313

[4] Lacouture ME, Patel AB, Rosenberg JE et al (2022) Management of dermatologic events associated with the nectin-4-directed antibody-drug conjugate enfortumab vedotin. Oncologist 27:e223–e232 35274723 10.1093/oncolo/oyac001 PMC8914492

[5] Rikitake Y, Mandai K, Takai Y (2012) The role of nectins in different types of cell-cell adhesion. J Cell Sci 125:3713–3722 23027581 10.1242/jcs.099572

[6] Sanders C, Lau JF, Dietrich D et al (2022) Nectin-4 is widely expressed in head and neck squamous cell carcinoma. Oncotarget. 13:1166–1173 36268557 10.18632/oncotarget.28299 PMC9584426

[7] Sethy C, Goutam K, Nayak D et al (2020) Clinical significance of a pvrl 4 encoded gene Nectin-4 in metastasis and angiogenesis for tumor relapse. J Cancer Res Clin Oncol 146:245–259 31617074 10.1007/s00432-019-03055-2 PMC11804762

[8] Tomiyama E, Fujita K, Rodriguez Pena MDC et al (2020) Expression of nectin-4 and PD-L1 in upper tract urothelial carcinoma. Int J Mol Sci. 21:5390 32751328 10.3390/ijms21155390 PMC7432817

[9] Minato A, Kimuro R, Ohno D et al (2023) Efficacy and tolerability of enfortumab vedotin for metastatic urothelial carcinoma: early experience in the real world. Anticancer Res 43:4055–4060 37648337 10.21873/anticanres.16594

[10] Seagen Inc., Astellas Pharma US, Inc. (2021) Padcev (enfortumab vedotinejfv) for injection [prescribing information]. Accessed 13 July 2021.

[11] Uemura K, Ito H, Jikuya R et al (2024) Enfortumab vedotin prolongs overall survival in metastatic urothelial carcinoma following pembrolizumab therapy in real-world data. Int J Urol 31:678–684 38402449 10.1111/iju.15437

[12] Eisenhauer EA, Therasse P, Bogaerts J et al (2009) New response evaluation criteria in solid tumours: revised RECIST guideline (version 1.1). Eur J Cancer 45:228–247 19097774 10.1016/j.ejca.2008.10.026

[13] Bellmunt J, Chouriri TK, Fourgeray R et al (2010) Prognostic factors in patients with advanced transitional cell carcinoma of the urothelial tract experiencing treatment failure with platinum-containing regimens. J Clin Oncol 28:1850–1855 20231682 10.1200/JCO.2009.25.4599

[14] National Cancer Institute. Cancer Therapy Evaluation Program: Protocol Development: Common Terminology Criteria for Adverse Events (CTCAE) v5.0. https://ctep.cancer.gov/protocolDevelopment/electronic_applications/ctc.htm#ctc_50 Accessed 27 November 2017.

[15] Kanda Y (2013) Investigation of the freely available easy-to-use software “EZR” for medical statistics. Bone Marrow Transplant 48:452–458 23208313 10.1038/bmt.2012.244 PMC3590441

[16] Matsubara N, Takahashi M, Kobayashi K et al (2023) Japanese subgroup analysis of EV-301: an open-label, randomized phase 3 study to evaluate enfortumab vedotin versus chemotherapy in subjects with previously treated locally advanced or metastatic urothelial carcinoma. Cancer Med 12:2761–2771 36052536 10.1002/cam4.5165 PMC9939146

[17] Hara T, Matsushita Y, Harada K et al (2024) Clinical outcomes in patients with advanced urothelial carcinoma treated with enfortumab vedotin: a retrospective multicenter study in Japan. Int J Urol 31:696–698 38424707 10.1111/iju.15435

[18] Takahashi S, Uemura M, Kimura T et al (2020) A phase I study of enfortumab vedotin in Japanese patients with locally advanced or metastatic urothelial carcinoma. Invest New Drugs 38:1056–1066 31444589 10.1007/s10637-019-00844-x PMC7340645

[19] Yu EY, Petrylak DP, O’Donnell PH et al (2021) Enfortumab vedotin after PD-1 or PD-L1 inhibitors in cisplatin-ineligible patients with advanced urothelial carcinoma (EV-201): a multicentre, single-arm, phase 2 trial. Lancet Oncol 22:872–882 33991512 10.1016/S1470-2045(21)00094-2

[20] Rosenberg JE, O’Donnell PH, Balar AV et al (2019) Pivotal trial of enfortumab vedotin in urothelial carcinoma after platinum and anti-programmed death 1/programmed death ligand 1 therapy. J Clin Oncol 37:2592–2600 31356140 10.1200/JCO.19.01140 PMC6784850

[21] Vlachou E, Matoso A, McConkey D et al (2023) Enfortumab vedotin-related cutaneous toxicity and radiographic response in patients with urothelial cancer: a single-center experience and review of the literature. Eur Urol Open Sci 49:100–103 36820243 10.1016/j.euros.2023.01.002 PMC9937876

[22] Duong TA, Valeyrie-Allanore L, Wolkenstein P et al (2017) Severe cutaneous adverse reactions to drugs. Lancet 390:1996–2011 28476287 10.1016/S0140-6736(16)30378-6

[23] Murata M, Ito T, Tanaka Y et al (2020) NECTIN4 expression in extramammary paget’s disease: implication of a new therapeutic target. Int J Mol Sci. 21:5891 32824340 10.3390/ijms21165891 PMC7460664

[24] Hirotsu KE, Rana J, Wnag JY et al (2021) Clinicopathologic characterization of enfortumab vedotin-associated cutaneous toxicity in patients with urothelial carcinoma. J Am Acad Dermatol 85:1610–1611 33301805 10.1016/j.jaad.2020.11.067

[25] Prieto-Torres L, Llamas-Velasco M, Machan S et al (2016) Taxanes-induced cutaneous eruption: another histopathologic mimicker of malignancy. J Eur Acad Dermatol Venereol 30:638–644 26558745 10.1111/jdv.13475

[26] Liu HB, Wu Y, Lv TF et al (2013) Skin rash could predict the response to EGFR tyrosine kinase inhibitor and the prognosis for patients with non-small cell lung cancer: a systematic review and meta-analysis. PLoS One 8:e55128 23383079 10.1371/journal.pone.0055128 PMC3559430

[27] Mendelsohn J, Baselga J (2003) Status of epidermal growth factor receptor antagonists in the biology and treatment of cancer. J Clin Oncol 21:2787–2799 12860957 10.1200/JCO.2003.01.504

[28] Lacouture ME (2006) Mechanisms of cutaneous toxicities to EGFR inhibitors. Nat Rev Cancer 6:803–812 16990857 10.1038/nrc1970

[29] Hayakawa N, Kikuchi E, Kaneko G et al (2024) Association between response to enfortumab vedotin and peripheral neuropathy in urothelial carcinoma patients: a multicenter retrospective study. Jpn J Clin Oncol. 10.1093/jjco/hyae082 38943559 10.1093/jjco/hyae082

[30] Taoka R, Kamada M, Izumi K et al (2024) Peripheral neuropathy and nerve electrophysiological changes with enfortumab vedotin in patients with advanced urothelial carcinoma: a prospective multicenter cohort study. Int J Clin Oncol 29:602–611 38418804 10.1007/s10147-024-02481-8

[31] Best RL, LaPointe NE, Azarenko O et al (2021) Microtubule and tubulin binding and regulation of microtubule dynamics by the antibody drug conjugate (ADC) payload, monomethyl auristatin E (MMAE): Mechanistic insights into MMAE ADC peripheral neuropathy. Toxicol Appl Pharmacol 421:115534 33852878 10.1016/j.taap.2021.115534

[32] Juan-Carlos PM, Herrera F, Blanco A et al (2021) ABC transporter superfamily. An updated overview, relevance in cancer multidrug resistance and perspectives with personalized medicine. Mol Biol Rep 48:1883–1901 33616835 10.1007/s11033-021-06155-w

[33] Wu H, Kang H, Liu Y et al (2013) Association of ABCB1 genetic polymorphisms with susceptibility to colorectal cancer and therapeutic prognosis. Pharmacogenomics 14:897–911 23746184 10.2217/pgs.13.78

[34] Chen R, Gupta S, O’Donnell PH et al (2015) CD30 downregulation, MMAE resistance, and MDR1 upregulation are all associated with resistance to brentuximab vedotin. Mol Cancer Ther 14:1376–1384 25840583 10.1158/1535-7163.MCT-15-0036 PMC4458438

[35] Miyake M, Nishimura N, Oda Y et al (2024) Enfortumab vedotin following platinum-based chemotherapy and immune checkpoint inhibitors for advanced urothelial carcinoma: response, survival and safety analysis from multicenter real-world Japanese cohort. Jpn J Clin Oncol 54:329–338 38061911 10.1093/jjco/hyad170

