# Supplementary material for: Treatment-related skin reactions in enfortumab vedotin as a surrogate marker of survival and treatment response
Source: Int J Clin Oncol. 2024 Dec 16;30(2):267–76. doi: 10.1007/s10147-024-02672-3 (PMC11785594; doi:10.1007/s10147-024-02672-3)

**Treatment-Related Skin Reactions in Enfortumab Vedotin as a Surrogate Marker of Survival and Treatment Response**

Jun Nagayama^1^, Satoshi Inoue^1^, Hiroki Sai^1,2^, Akira Hayakawa^3^, Yuri Yuguchi^4^, Tomohide Suzuki^5^, Hirotaka Matsui^6^, Takuma Yuba^7^, Koya Morishita^8^, Shusuke Akamatsu¹

1. Department of Urology, Nagoya University Graduate School of Medicine
2. Department of Urology, Japanese Red Cross Aichi Medical Center Nagoya Daiichi Hospital
3. Department of Urology, Yokkaichi Municipal Hospital
4. Department of Urology, Chukyo Hospital
5. Department of Urology, Nagoya Medical Center
6. Department of Urology, Toyohashi Municipal Hospital
7. Department of Urology, Kariya Toyota General Hospital
8. Department of Urology, Komaki Municipal Hospital

Corresponding author: Jun Nagayama M.D., E-mail: j.nagayama@med.nagoya-u.ac.jp, ORCID ID: 0000-0001-6239-2917 Department of Urology, Nagoya University Graduate School of Medicine, 65 Tsuruma-cho, Showa-Ku, Nagoya, Aichi, 466-8550, Phone number: TEL 81-52-744-2985, FAX 81-52-744-2319

**Supplementary Table 1. Comparison of laboratory data between TRSRs and non-TRSRs**

| Variables | **TRSRs**  (n = 34) | **non-TRSRs**  (n = 33) | ***p*** |
| --- | --- | --- | --- |
| Albumin, g/dL (SD) | 3.62 ± 0.49 | 3.47 ± 0.43 | 0.181 |
| C-reactive protein, mg/L (IQR) | 14.7 (3.2­­­­­­­­–42.0) | 11.2 (3.5–32.3) | 0.861 |
| Creatinine, mg/dL (IQR) | 1.08 (0.84–1.33) | 1.07 (0.89–1.42) | 0.531 |
| eGFR, mL/min/1.73m² (IQR) | 52.0 (39.5–59.4) | 49.8 (38.0–60.6) | 0.711 |
| Lactate dehydrogenase, U/L (IQR) | 198.0 (170.8–246.8) | 197.0 (174.0–257.0) | 0.851 |
| Hemoglobin, g/dL (SD) | 11.5 ± 1.78 | 10.6 ± 1.90 | **0.040** |
| Platelet, 10^4^/μL (IQR) | 26.2 (20.3–30.6) | 22.1 (19.2–38.4) | 0.625 |
| Neutrophil, /μL (IQR) | 4500 (3580–5510) | 4481 (3551–6552) | 0.832 |
| Monocyte, /μL (IQR) | 560 (345–738) | 580 (424.5–837.5) | 0.318 |

Abbreviations

IQR: interquartile range

SD: standard deviation

eGFR: estimated glomerular filtration rate

TRSRs: treatment-related skin reactions

**Supplementary Table 2. Patients’ characteristics with and without dose reduction and discontinuation of EV due to any TRAEs among overall patients**

| Variables | **with dose reduction**  **and discontinuation**  (n = 25) | **without dose reduction and discontinuation**  (n = 42) | ***p*** |
| --- | --- | --- | --- |
| Age median, years (IQR) | 73.0 (67.0–79.0) | 71.0 (68.0–75.0) | 0.599 |
| Sex, no. (%)  Male  Female | 21 (84.0)  4 (16.0) | 32 (76.2)  10 (23.8) | 0.544 |
| ECOG PS, no. (%)  0–1  2 | 24 (96.0)  1 (4.0) | 37 (88.1)  5 (11.9) | 0.399 |
| Bellmunt risk score^†^, no. (%)  0–1  ≥2 | 16 (64.0)  9 (36.0) | 23 (54.8)  19 (45.2) | 0.609 |
| Primary site, no. (%)  Bladder  Upper urinary tract  Bladder + upper urinary tract | 17 (68.0)  8 (32.0)  0 (0.0) | 22 (52.4)  13 (31.0)  7 (16.7) | 0.092 |
| Metastatic site, no. (%)  Lymph node only  Visceral  Liver | 9 (36.0)  16 (64.0)  8 (32.0) | 10 (23.8)  32 (76.2)  10 (23.8) | 0.401  0.571 |
| Line of therapy, no. (%)  3  ≥4 | 19 (76.0)  6 (24.0) | 29 (69.0)  13 (31.0) | 0.588 |
| The most recent systemic therapy  before EV, no. (%)  ICI  Chemotherapy | 21 (84.0)  4 (16.0) | 34 (81.0)  8 (19.0) | 1.000 |
| Prior chemotherapy, cycles (IQR) | 4 (3–6) | 4 (2–5.25) | 0.918 |
| Prior ICI, cycles (IQR) | 8.5 (4–15.75) | 5.5 (4–9) | 0.180 |
| Duration of prior therapy to EV,  months (IQR) | 0 (0–1) | 0 (0–1) | 0.595 |
| EV, cycles (IQR) | 6 (4–11) | 4 (2–7) | 0.057 |
| Dose reduction due to TRAEs, no. (%)  one-step reduction (1.0mg/kg)  two-step reduction (0.75mg/kg) | 17 (68.0)  13  4 |  |  |
| Discontinuation due to TRAEs, no. (%) | 10 (40.0) |  |  |
| Grade≥3 TRAEs, no. (%) | 9 (36.0) | 6 (14.3) | 0.067 |
| Prior radical therapy, no. (%)  Yes  No | 13 (52.0)  12 (48.0) | 27 (64.3)  15 (35.7) | 0.440 |
| Variant histology, no. (%) | 1 (6.2) | 2 (5.9) | 1.000 |

†Bellmunt risk score- liver metastases, hemoglobin level < 10g/dL, and ECOG PS > 0 are the factors;

every point is added for each factor.

Abbreviations:

IQR: interquartile range

ECOG PS: Eastern Cooperative Oncology Group performance status

EV: enfortumab vedotin

ICI: immune-checkpoint inhibitor

TRAEs: treatment-related adverse events

**Supplementary Table 3. Patients’ characteristics with and without dose reduction and discontinuation of EV due to any TRSRs among overall patients**

|  | **With dose reduction**  **and discontinuation**  (n = 17) | **Without dose reduction**  **and discontinuation**  (n = 17) | ***p*** |
| --- | --- | --- | --- |
| Age median, years (IQR) | 73.0 (67.0–78.0) | 70.0 (62.0–75.0) | 0.490 |
| Sex, no. (%)  Male  Female | 14 (82.4)  3 (17.6) | 12 (70.6)  5 (29.4) | 0.688 |
| ECOG PS, no. (%)  0–1  2 | 16 (94.1)  1 (5.9) | 16 (94.1)  1 (5.9) | 1.000 |
| Bellmunt risk score^†^, no. (%)  0–1  ≥2 | 12 (70.6)  5 (29.4) | 11 (64.7)  6 (35.3) | 1.000 |
| Primary site, no. (%)  Bladder  Upper urinary tract  Bladder + Upper urinary tract | 10 (58.8)  7 (41.2)  0 (0.0) | 8 (47.1)  7 (41.2)  2 (11.8) | 0.632 |
| Metastatic site, no. (%)  Lymph node only  Visceral  Liver | 4 (23.5)  13 (76.5)  7 (41.2) | 5 (29.4)  12 (70.6)  3 (17.6) | 1.000  0.259 |
| Line of therapy, no. (%)  3  ≥4 | 14 (82.4)  3 (17.6) | 12 (70.6)  5 (29.4) | 0.688 |
| The most recent systemic therapy  before EV, no. (%)  ICI  Chemotherapy | 16 (94.1)  1 (5.9) | 15 (88.2)  2 (11.8) | 1.000 |
| Prior chemotherapy, cycles (IQR) | 4 (2–4) | 3.5 (0–4.25) | 0.646 |
| Prior ICI, cycles (IQR) | 8.5 (4–16.75) | 6 (4–9) | 0.199 |
| Duration of prior therapy to EV,  months (IQR) | 0 (0–0) | 0 (0–2) | 0.071 |
| EV, cycles (IQR) | 10 (6–11) | 5 (4–9) | 0.097 |
| Grade 3 TRSRs, no. (%) | 6 (35.3) | 1 (5.9) | 0.085 |
| Prior radical therapy, no. (%)  Yes  No | 10 (58.8)  7 (41.2) | 11 (64.7)  6 (35.3) | 1.000 |
| Variant histology, no. (%) | 1 (8.3) | 0 (0.0) | 1.000 |

†Bellmunt risk score- liver metastases, hemoglobin level < 10g/dL, and ECOG PS > 0 are the factors;

every point is added for each factor.

Abbreviations:

IQR: interquartile range

ECOG PS: Eastern Cooperative Oncology Group performance status

EV: enfortumab vedotin

ICI: immune-checkpoint inhibitor

TRSRs: treatment-related skin reactions

**Supplementary Figure 1. Kaplan-Meier curves of CSS and PFS in comparison with and without dose reduction/treatment discontinuation due to any TRAEs**


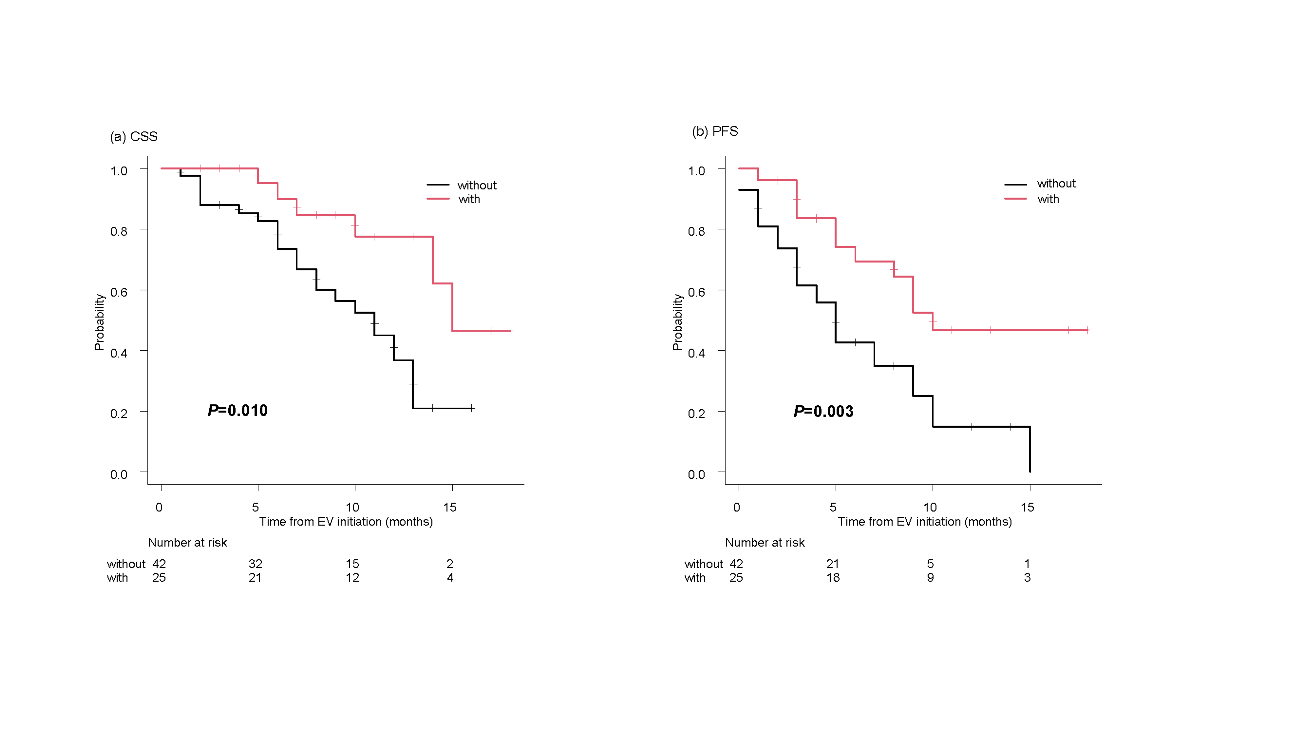


**Supplementary Figure 2. Kaplan-Meier curves of CSS and PFS in comparison with and without dose reduction/treatment discontinuation due to TRSRs**


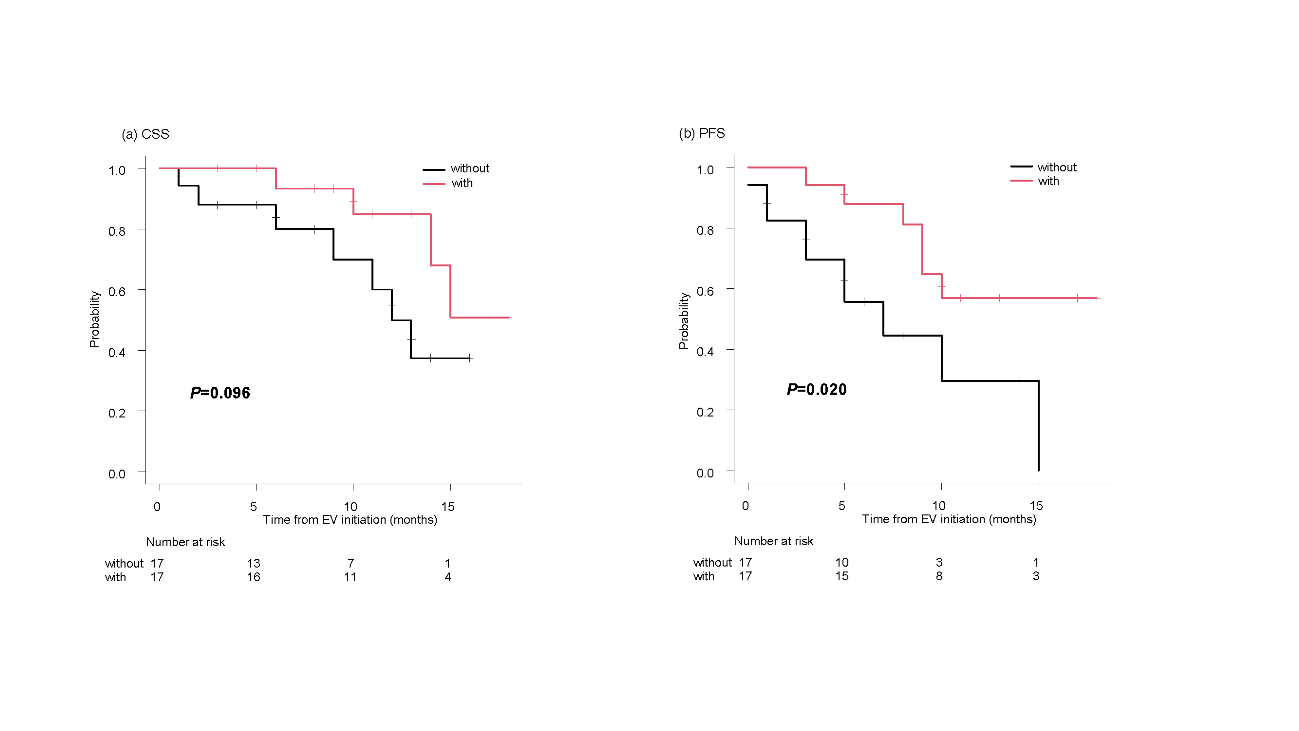

Supplement: Supplementary file 1 — Supplementary file1 (DOCX 240 KB) [file 10147_2024_2672_MOESM1_ESM.docx]
